# Slc25a39 and Slc25a40 Expression in Mice with Bile Duct Ligation or Lipopolysaccharide Treatment

**DOI:** 10.3390/ijms23158573

**Published:** 2022-08-02

**Authors:** Atsushi Kawase, Momoko Hatanaka, Naoya Matsuda, Hiroaki Shimada, Masahiro Iwaki

**Affiliations:** 1 Department of Pharmacy, Faculty of Pharmacy, Kindai University, 3-4-1 Kowakae, Higashiosaka 577-8502, Osaka, Japan; 1911610045k@kindai.ac.jp (M.H.); 1711610004k@kindai.ac.jp (N.M.); shimada@phar.kindai.ac.jp (H.S.); kindai20@gmail.com (M.I.); 2 Pharmaceutical Research and Technology Institute, Kindai University, 3-4-1 Kowakae, Higashiosaka 577-8502, Osaka, Japan; 3 Antiaging Center, Kindai University, 3-4-1 Kowakae, Higashiosaka 577-8502, Osaka, Japan

**Keywords:** cholestasis, glutathione, oxidative stress, mitochondria, endotoxin

## Abstract

SLC25A39/40, involved in mitochondrial GSH (mGSH) import from the cytoplasm, is essential for protection against oxidative stress and mitochondrial dysfunction. We examined the effects of cholestasis, through bile duct ligation (BDL) and lipopolysaccharide (LPS)-induced inflammation in mice, on Slc25a39/40 expression. Additionally, we used human clear cell renal carcinoma (KMRC-1) cells to elucidate the mechanism of regulation of SLC25A39/40 expression in the kidneys after LPS treatment. BDL resulted in a decrease in *Slc25a39* mRNA in the liver and a decrease in *Slc25a39/40* mRNA and protein in the kidneys. Consequently, there was a significant decrease in mGSH levels in the kidneys of BDL mice compared with those in sham mice. LPS treatment resulted in increased *Slc25a40* expression in the kidneys. In KMRC-1 cells, the combination treatment of LPS-RS or FPS-ZM1 with LPS suppressed the LPS-induced increase in *SLC25A40*, suggesting that *SLC25A40* expression could be regulated by the signaling pathway via toll-like receptor 4 and the receptor for advanced glycation end products, respectively. Our findings contribute to understanding the role of mGSH in the maintenance of the mitochondrial redox state. To the best of our knowledge, this is the first study that demonstrates the changes in Slc25a39/40 expression in mice with cholestasis-associated renal injury and LPS-induced inflammation.

## 1. Introduction

Intracellular GSH (γ-glutamyl-L-cysteinyl-glycine) is crucial in the maintenance of normal cell function. It performs several functions, including protection against oxidative stress, cellular proliferation, and cell division. It is distributed in the mitochondria (10−15%), nucleus, and endoplasmic reticulum [1]. A net negative charge of GSH at physiological pH indicates the presence of a transporter for its uptake into the mitochondria [2,3]. However, such a transporter has not been identified yet. Wang et al. revealed that *SLC25A39* and its paralogue *SLC25A40* (*SLC25A39/40*) are required for mitochondrial GSH (mGSH) import [4]. Additionally, the *SLC25A39/40* double-knockout Jurkat cells are incapable of cell proliferation [4]. The mGSH import is important to maintain cell function while avoiding mitochondrial dysfunction [5,6].

Cholestasis is characterized by a decrease in bile secretion or flow from hepatocytes. The accumulation of bile acid (BA) in the liver is induced by cholestasis. Cholestasis-associated oxidative stress and inflammation have a significant impact on the levels of intracellular GSH [7,8,9,10]. GSH is involved in reactive oxygen species scavenging and is converted into glutathione disulfide (GSSG) under oxidative stress, resulting in a decreased GSH/GSSG ratio [11]. The fall in mGSH content that is caused by cholestasis is speculated to reflect the changes in the mitochondrial redox state. Therefore, the impact of cholestasis on mGSH import, via alterations in *SLC25A39/40* expression, needs to be clarified. Cholestasis leads to impaired physiological functions of various organs besides that of the liver. In particular, kidney functions are susceptible to changes during cholestasis, and this cholestasis-associated renal injury is called ‘cholemic nephropathy’ [12,13,14]. However, the alterations in SLC25A39/40 expression during cholemic nephropathy have not been studied yet.

In addition to inflammation with cholestasis, lipopolysaccharide (LPS)-induced inflammation has been widely examined to clarify the effects of oxidative stress and inflammation on transporters in the liver and kidneys [15,16,17]. LPS binds to the toll-like receptor (TLR) 4, following the activation of the NF-κB signaling pathway, and induces an acute inflammatory condition in mice. The interplay between TLR4 and the receptor for advanced glycation end products (RAGE) is involved in the propagation and amplification of inflammatory responses [18]. The initial responses of cholestatic inflammation and LPS-induced inflammation during their induction show large differences, which may be the reason behind the different effects of inflammation on SLC25A39/40 expression.

This prompted us to examine the effects of cholestasis using bile duct ligation (BDL) and LPS treatment on SLC25A39/40 expression and GSH levels in the liver and kidneys. We used mice as the animal model for cholestasis induced by BDL and LPS-induced inflammation. We also examined whether BDL- and LPS-induced inflammation affected mGSH levels. It is well known that the kidneys of BDL mice are inflamed extrahepatic organs [13,19]. In addition, we conducted an in vitro study using human clear cell renal carcinoma (KMRC-1) cells to clarify the mechanism of regulation of *SLC25A39/40* expression in the kidneys following LPS treatment.

## 2. Results

### 2.1. Slc25a39/40 Expression in Mice with BDL

**Figure 1:**
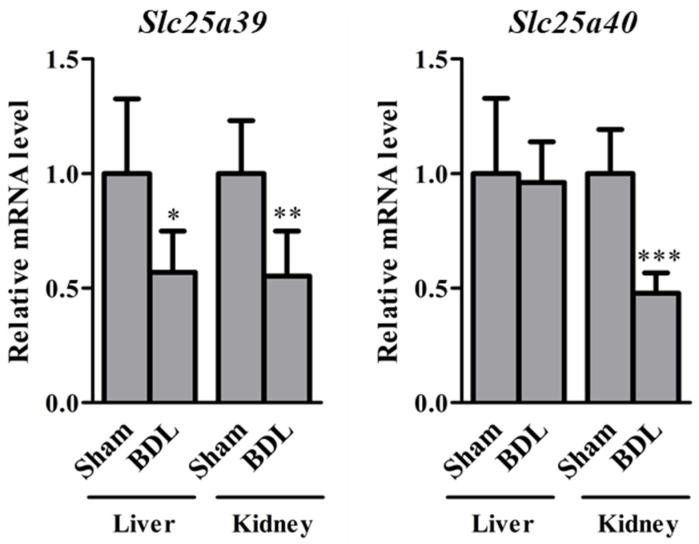
Relative mRNA levels of *Slc25a39/40* in the liver and kidneys of sham and bile duct ligation (BDL) mice. The results are expressed as the mean ± S.D. of each group (n = 5). Significant differences (* *p* < 0.05, ** *p* < 0.01, and *** *p* < 0.001) between sham and BDL mice were observed.

**Figure 2:**
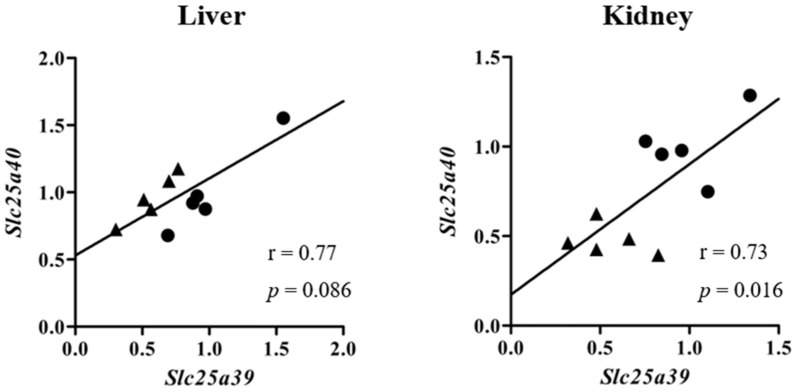
Correlation between relative mRNA levels of *Slc25a39* and *Slc25a40* in the liver and kidneys of sham and bile duct ligation (BDL) mice. Closed circles and triangles represent sham and BDL mice, respectively.

**Figure 3:**
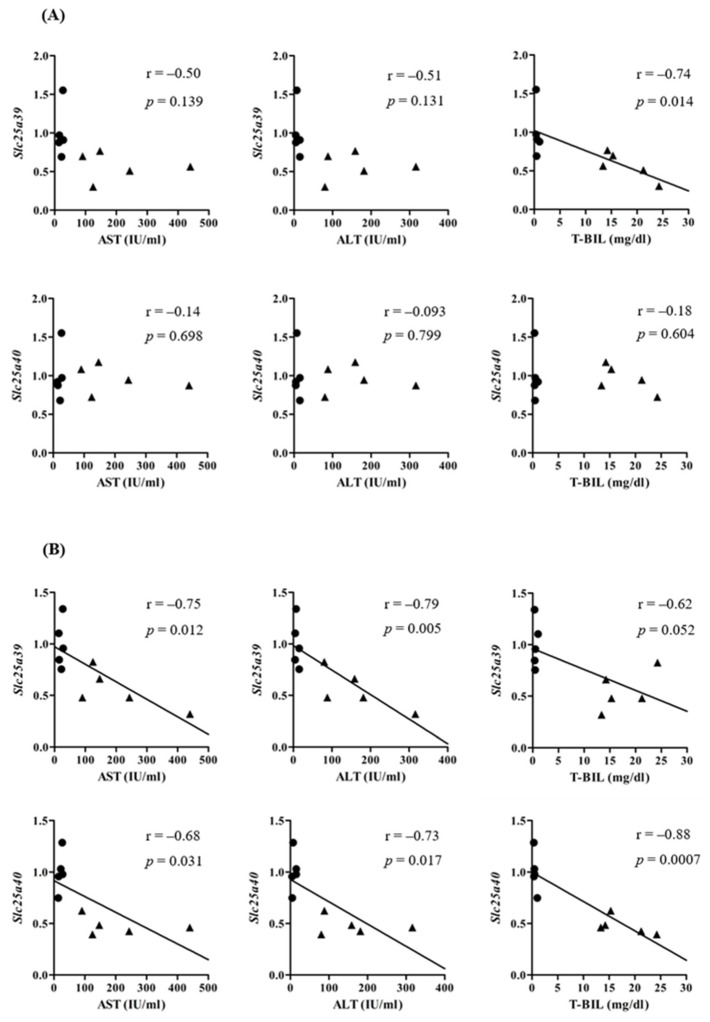
Correlation between aspartate aminotransferase (AST; IU/mL), alanine aminotransferase (ALT; IU/mL), or total bilirubin (T-BIL; mg/dL), and relative mRNA levels of *Slc25a39/40* in the liver (**A**) and kidneys (**B**) of sham and bile duct ligation (BDL) mice. Closed circles and triangles represent sham and BDL mice, respectively.

**Figure 4:**
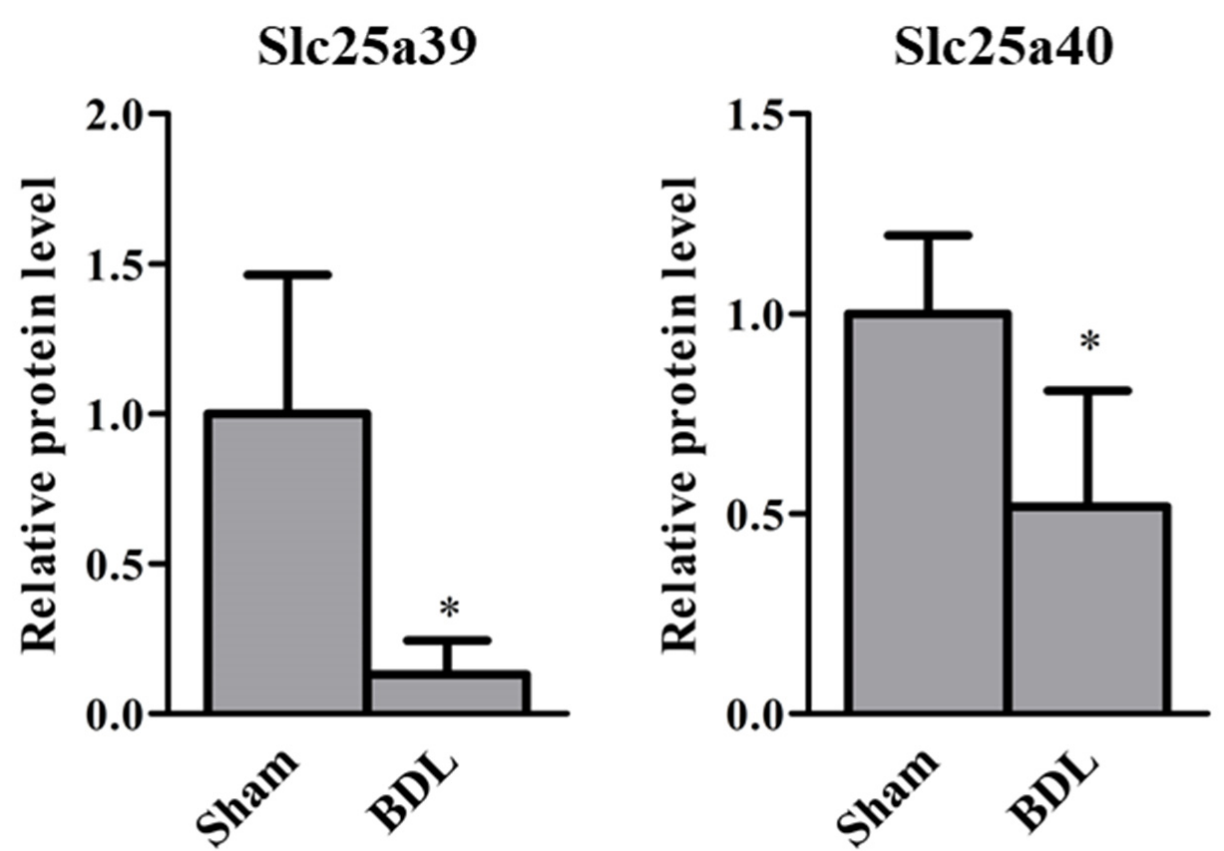
Relative protein levels of Slc25a39/40 in the kidneys of sham and bile duct ligation (BDL) mice. The results are expressed as the mean ± S.D. of each group (n = 3−4). Significant differences (* *p* < 0.05) between sham and BDL mice were observed.

Relative mRNA levels of *Slc25a39/40* in the liver and kidneys of sham and BDL mice were determined (Figure 1). *Slc25a39* mRNA levels in the liver and kidneys and *Slc25a40* mRNA levels in the kidneys of BDL mice were significantly lower than those of sham mice. The correlation between relative mRNA levels of *Slc25a39* and *Slc25a40* in the liver and kidneys of sham and BDL mice is depicted in Figure 2. Positive correlations were observed between *Slc25a39* and *Slc25a40* mRNA levels in the liver as well as the kidneys. In particular, the kidneys showed a significant positive correlation. The correlations between the mRNA levels of *Slc25a39/40* and the biochemical markers in BDL, such as aspartate aminotransferase (AST), alanine aminotransferase (ALT), or total bilirubin (T-BIL), was also determined (Figure 3). The plasma levels of AST, ALT, and T-BIL in sham and BDL mice were shown (Figure S1). In the liver of sham and BDL mice, a significant negative correlation was observed between the T-BIL level and the *Slc25a39* mRNA level. The kidneys of sham and BDL mice showed a significant correlation between *Slc25a39/40* and the parameters AST, ALT, and T-BIL, suggesting that plasma biochemical markers such as AST, ALT, and T-BIL could be indicators of *Slc25a39/40* expression levels in the kidneys. We investigated whether the Slc25a39/40 protein expression in the kidneys was altered similarly to the mRNA expression levels (Figure 4). Relative protein levels of Slc25a39/40 in the kidneys of BDL mice were significantly decreased compared with those of sham mice.

### 2.2. GSH Levels in Mice with BDL

**Figure 5:**
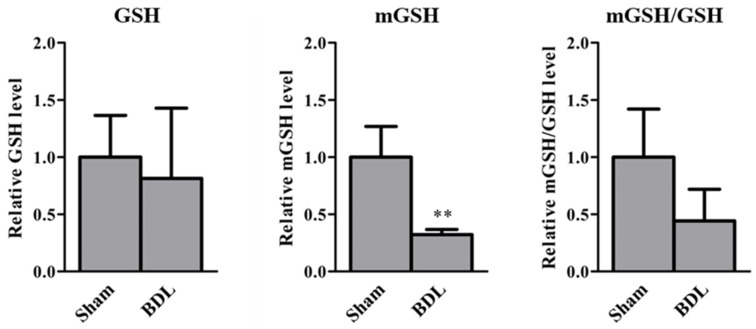
Relative glutathione (GSH), mitochondrial GSH (mGSH), and mGSH/GSH levels in the kidneys of sham and bile duct ligation (BDL) mice. The results are expressed as the mean ± S.D. of each group (n = 3−4). Significant differences (** *p* < 0.01) between sham and BDL mice were observed.

Figure 5 illustrates the relative GSH, mGSH, and mGSH/GSH levels in the kidneys of sham and BDL mice. The relative mGSH levels in BDL mice were significantly decreased compared with those in sham mice, whereas there was no difference in the relative GSH levels in the S9 fraction between the sham and BDL mice. The ratios of mGSH to GSH of BDL mice tended to decrease (*p* = 0.10).

### 2.3. Slc25a39/40 Expression in Mice with LPS-Induced Inflammation

**Figure 6:**
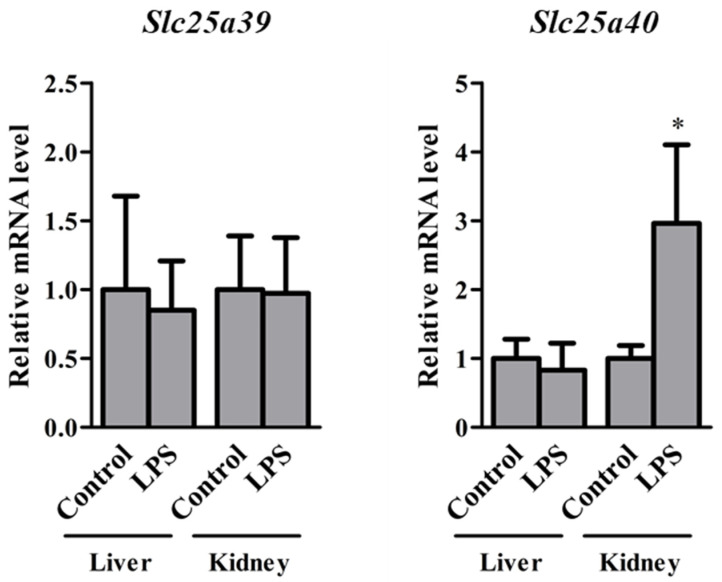
Relative mRNA levels of *Slc25a39/40* in the liver and kidneys of control and lipopolysaccharide (LPS) mice 24 h after vehicle or LPS administration. The results are expressed as the mean ± S.D. of each group (n = 4−5). Significant differences (* *p* < 0.05) between control and LPS mice were observed.

**Figure 7:**
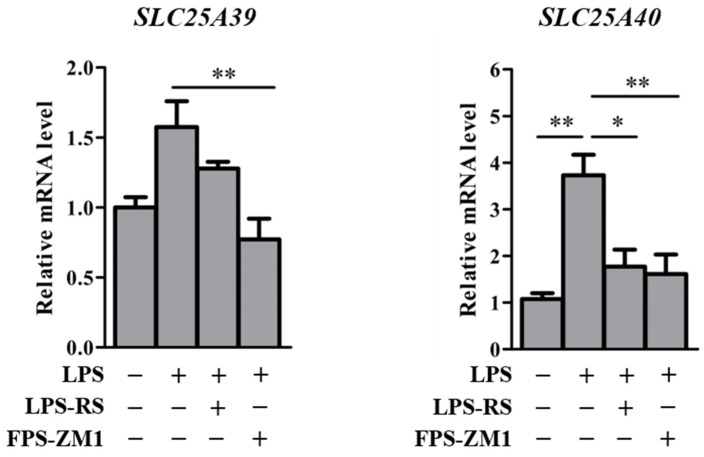
Relative mRNA levels of *SLC25A39/40* in the KMRC-1 cells 24 h after treatments of vehicle lipopolysaccharide (LPS) alone (1 μg/mL), or the combination of LPS-RS (1 μg/mL) or FPS-ZM1 (1 μg/mL) and LPS (1 μg/mL) to KMRC-1 cells. The results are expressed as the mean ± S.E. of each group (n = 3−5). Significant differences (* *p* < 0.05 and ** *p* < 0.01) were observed.

To clarify whether LPS-induced inflammation affects *Slc25a39/40* expression, the relative mRNA levels of *Slc25a39/40* in the liver and kidneys of control and LPS mice were determined (Figure 6). Control and LPS mice had similar *Slc25a39* mRNA levels in the liver and kidneys. The *Slc25a40* mRNA levels in the kidneys, but not in the liver, of LPS mice were significantly increased compared with those of control mice. To determine the regulatory mechanisms of *SLC25A39/40* after LPS treatment, an in vitro study was carried out (Figure 7). Relative mRNA levels of *SLC25A39/40* in the KMRC-1 cells were examined 24 h after being treated with the vehicles: LPS alone, LPS + LPS-RS, and LPS + FPS-ZM1. We used LPS-RS as a TLR4 antagonist and FPS-ZM1 as a RAGE antagonist to clarify the involvement of TLR4 and RAGE in the transcriptional regulation of the *SLC25A39/40* gene. Following LPS treatment, the mRNA levels of *SLC25A39* were significantly decreased compared with those in the control when combined with FPS-ZM1, but not with LPS-RS. LPS treatment resulted in significant increases in *SLC25A40* mRNA in KMRC-1 cells. The *SLC25A40* mRNA levels of LPS treatment were significantly decreased compared with the control levels by combination with both LPS-RS and FPS-ZM1.

## 3. Discussion

Our study revealed that the expression of Slc25a39/40 changes in mice with cholestasis and LPS-induced inflammation. Following a BDL operation or LPS treatment in mice, the GSH levels and *Slc25a39/40* expression in the liver and kidneys were examined. The BDL operation and LPS treatment resulted in a decrease in *Slc25a39/40* expression in the kidneys and an increase in *Slc25a40* expression, respectively. In particular, *Slc25a39/40* mRNA levels in the kidneys, but not liver, of BDL mice correlated with the plasma levels of AST, ALT, and T-BIL. Wang et al. found that Slc25a39/40 was involved in GSH transport into the mitochondria [4], which helps us to understand its role in the maintenance of the mitochondrial redox state by mGSH. To our knowledge, the present study is the first to examine the changes in *Slc25a39/40* expression in mice with cholestasis-associated injury and LPS-induced inflammation.

Changes in the expression of uptake and efflux transporters would prevent the accumulation of potentially toxic biliary compounds such as bile acids in BDL mice. BAs, such as taurocholic acid and β-muricholic acid, induce proinflammatory mediators in hepatocytes [20]. The plasma concentrations of proinflammatory cytokines, such as tumor necrosis factor-α and interleukin-1β, were increased by BDL via the accumulation of dendritic cells in the liver [21,22]. The plasma bilirubin levels were also increased in the BDL mice [23]. A combination of these factors (BA, proinflammatory cytokines, and bilirubin) could affect the transporter expression in the extrahepatic organs of BDL mice. It is well known that the kidneys of BDL mice are inflamed extrahepatic organs [13,19]. The concentrations of blood urea nitrogen and creatinine in serum, and glucose, T-BIL, protein, alkaline phosphatase, gamma-glutamyl transpeptidase, and bile acids in urine of BDL mice were higher compared with those of sham mice [19]. The inflammation in extrahepatic organs such as kidneys was induced by oxidative stress following mitochondrial impairment [24,25,26]. Further studies of the renal inflammatory conditions of BDL mice are needed in future studies, because it is necessary to clarify the correlation between renal inflammation and Slc25a39/40 expression levels in the kidneys of BDL mice. Both BA and bilirubin act as ligands for nuclear receptors that regulate the transcription of transporters. BA binds to the nuclear receptors pregnane X receptor (NR1I2) and farnesoid X receptor (NR1H4), and bilirubin binds to the constitutive androstane receptor (NR1I3) [27]. Consequently, BDL shows adaptive changes in the levels of transporters: a decrease in Ntcp in the liver, and an increase in Mrp in the liver and kidneys [28,29]. The mRNA levels of *Oatp1a1* (Slco1a1) and *Oatp1a2* (Slco1a2) decreased significantly in the kidneys of BDL mice [30]. However, the detailed mechanisms of transcriptional regulation of *Slc25a39/40* are unclear. Further studies are needed to elucidate the *Slc25a39/40* regulation responsible for its role in protecting the mitochondria from oxidative stress.

The *Slc25a39/40* mRNA levels in the kidneys of sham and BDL mice correlated significantly with the plasma AST, ALT, and T-BIL levels (Figure 3B), suggesting that these parameters can be potential indicators of the variation of *Slc25a39/40* expression and mGSH levels in the kidneys. Wang et al. demonstrated that the amount of GSH uptake into the mitochondria was six times higher with *Slc25a39* expression [4]. The effects of BDL on GSH levels in kidneys are debatable. Some studies reported that the renal GSH content was significantly decreased [31], while others have shown that it was significantly increased [32]. The type of species or strain could have influenced the effect of BDL on *Slc25a39/40* expression, although the precise cause of these contradicting results is unclear. We have shown decreased *Slc25a39/40* expression in the kidneys of BDL mice following the reduction in relative mGSH and mGSH/GSH levels, without changes of GSH in the S9 fraction (Figure 4 and Figure 5). The correlation between the protein levels of Slc25a39/40 and mGSH in the kidney was examined. The significant correlation (r = 0.77, *p* = 0.042) between Slc25a39 and mGSH was observed. Slc25a40 showed the correlation (r = 0.69, *p* = 0.088) for mGSH. In addition to total intracellular GSH, the distribution of GSH to each organelle needs to be evaluated, because the mGSH levels of BDL mice were significantly decreased when compared with those of sham mice, without changes in the GSH levels in the S9 fraction. Additionally, the influence of the species and strain on the effects of BDL on the expression and function of *SLC25A39/40* should be evaluated.

In mice with LPS-induced inflammation, the *Slc25a40* mRNA levels in the kidneys, but not in the liver, were significantly increased (Figure 6). These changes in *Slc25a39/40* were markedly different from those in BDL mice. LPS treatment has been reported to affect the expression levels of some transporters in the kidneys. For example, LPS treatment increased *Mdr1b* mRNA levels in the kidneys significantly, while the increase in the liver was not significant [16]. TLR4 and RAGE receptors for LPS functionally interact to regulate inflammatory responses [33,34]. The *SLC25A40* mRNA levels were significantly increased in both the kidneys of LPS mice and KMRC-1 cells after LPS treatment, when compared with those in the controls (Figs. 6 and 7). The inhibition of TLR4 or RAGE decreased the *SLC25A40* mRNA levels in KMRC-1 cells after LPS treatment, compared to the control levels (Figure 7). There was a significant difference in *SLC25A39* mRNA levels between LPS and LPS + FPS-ZM1. *SLC25A39* mRNA levels in KMRC-1 cells treated with LPS + FPS-ZM1 were significantly lower when compared with those with LPS treatment alone, although the induction of *SLC25A39* mRNA in KMRC-1 cells after LPS treatment was not significant. These results suggest that the transcriptional regulation of *SLC25A39* is partly regulated by a signaling pathway via RAGE. Although it has been suggested that TLR4 and RAGE are partly involved in the increased expression of SLC25A39/40 after LPS treatment, it is unclear which signaling molecules are associated with SLC25A39/40 regulation. Further studies by Western blot analysis using antibodies against specific components of TLR4 and RAGE signaling cascades are needed to clarify the signaling molecules.

Our previous published and unpublished results demonstrate that the changes in transporter expression in the inflamed kidneys are more remarkable than those in the inflamed liver [35]. It is suggested that the expression of Slc25a39/40 in kidneys is susceptible to alteration during inflammation. Elucidation of the detailed mechanism of Slc25a39/40 regulation will lead to clarification of the relationship between intracellular GSH transport and the biological defense system.

## 4. Materials and Methods

### 4.1. Chemicals and Reagents

GSH, GSSG, Sepasol-RNA I Super G, and Dulbecco’s modified Eagle’s medium (DMEM) were purchased from Nacalai Tesque Inc. (Kyoto, Japan). ReverTra Ace was procured from Toyobo Co., Ltd. (Osaka, Japan). Fast SYBR Green Master Mix and the BCA protein assay kit were purchased from Thermo Fisher Scientific Inc. (Waltham, MA, USA). Transaminase CII-test Wako and MS-grade porcine pancreatic trypsin were obtained from Fujifilm Wako Pure Chemical Co., Ltd. (Osaka, Japan). The QuantiChrom bilirubin assay kit was obtained from BioAssay Systems, LLC. (Hayward, CA, USA). Percoll was obtained from Cytiva (Tokyo, Japan). LPS from *Escherichia coli* was procured from Sigma-Aldrich Co., LLC. (St. Louis, MO, USA). LPS-RS was obtained from InvivoGen Inc. (San Diego, CA, USA). FPS-ZM1 was purchased from Cayman Chemical Co. (Ann Arbor, MI, USA). Oligonucleotide primers were obtained from Eurofins Genomics Inc. (Luxembourg, Luxembourg). All other chemicals and solvents were of MS grade or higher and of commercially available purity.

### 4.2. Animals and Treatments

Male ICR mice, 5–6 weeks old, were purchased from Japan SLC Inc. (Shizuoka, Japan). The mice were housed in a climate-controlled room at 24 ± 2 °C with relative humidity of 55 ± 10% and a 12 h lighting schedule (7:00 a.m. to 7:00 p.m.), and were allowed free access to standard laboratory chow (MF; Oriental Yeast Co., Ltd., Tokyo, Japan). For the BDL study, mice were randomly divided into two groups: the sham group and the BDL group. Mice in both groups were anesthetized with pentobarbital (50 mg/kg, i.p.). An incision of approximately 2 cm was made in the abdomen of mice in the BDL group using micro-dissecting scissors, and the bile duct was exposed and doubly ligated with 4-0 silk. The bile duct of sham-operated animals was exposed, but not ligated (control mice). After the surgery, the skin was sutured and the mice were maintained as described earlier. The sham-operated and BDL mice were euthanized after 5 days, and their liver and kidneys were removed. In the LPS group, the mice were treated with LPS (5 mg/kg, i.p.). The control group mice were treated with saline as a vehicle. The liver, kidneys, and blood of the control and LPS mice were removed 24 h after LPS injection, after subjecting them to euthanasia. All heparinized blood samples were centrifuged immediately at 1000× *g* for 10 min at 4 °C, and plasma was collected. Each sample was flash frozen in liquid nitrogen and subsequently stored at −80 °C until use. The study protocol was approved by the Committee for the Care and Use of Laboratory Animals of the Faculty of Pharmacy of Kindai University (Osaka, Japan).

### 4.3. Determination of mRNA Levels of Slc25a39/40 Using Real-Time Reverse Transcription-Polymerase Chain Reaction

Total RNA was extracted from the liver and kidneys of sham and BDL mice; the liver and kidneys of control and LPS mice, and KMRC-1 cells using Sepasol RNA I Super G. The RNA was then reverse transcribed into complementary DNA using ReverTra Ace qPCR RT Master Mix. The PCR mixtures were incubated at 95 °C for 10 s and then amplified at 95 °C for 5 s, 55 °C for 20 s, and 72 °C for 20 s for 40 cycles using Fast SYBR Green Master Mix. The oligonucleotide sequences (5′–3′) of the primers used for each mRNA target are as follows: *mSlc25a39*: TCTTGGCCCCATTCATTCCTTG and GTACCCCAAGACACTACAGCCAC, *mSlc25a40*: TGGAGCCTGAAACTGAAGGG and GAATGGGTTGTTCTGGGCCT, *mβ-actin*: GATCAAGATCATTGCTCCTCCTG and GCAGCTCAGTAACAGTCCGC, *hSLC25A39*: TGCCCTTCTCAGCCCTGTA and CACAAAGCTCATGCCCACAG, *hSLC25A40*: ACCCACTCCCCAAAGGAAAATG and GTTTGTTGCCTCCCTCTTCAC, and *hβ-actin*: CACCATTGGCAATGAGCGGTTC and AGGTCTTTGCGGATGTCCACGT. The data obtained were analyzed using the Step One Real-Time PCR System (Thermo Fisher Scientific, Inc.) and multiplex comparative method. The target mRNA levels were normalized to those of β-actin.

### 4.4. Plasma AST, ALT, and T-BIL Levels

Plasma AST and ALT levels were determined using the transaminase CII-test Wako. Plasma from sham and BDL mice (20 μL) was added to 500 μL of AST or ALT substrates and incubated for 5 min at 37 °C, following which chromogenic substrates were added to the reaction solutions. Absorbance was measured at 540 nm using an absorption spectrometer (Sunrise R, TECAN Group Ltd., Mannedorf, Switzerland). The AST and ALT activity were estimated using standard curves. Plasma T-BIL levels were determined using the QuantiChrom bilirubin assay kit. Plasma from sham and BDL mice (50 μL) was added to 200 μL of working reagent. After incubation for 10 min, absorbance at 540 nm was measured using an absorption spectrometer (Sunrise R).

### 4.5. Isolation of Mitochondria

The mitochondria were isolated using the method described in previous reports [36,37], with minor modifications. The kidneys were homogenized with 5% trichloroacetic acid containing 0.5 mM ethylenediaminetetraacetic acid (EDTA) to minimize the GSH oxidation or degradation. The homogenates were centrifuged at 740× *g* for 5 min at 4 °C and the supernatant was collected, then further centrifuged at 9000× *g* for 30 min at 4 °C and the resulting supernatant and precipitate, which were designated as the S9 fraction and crude mitochondrial fraction, respectively, were collected. Pure mitochondrial fraction (Mit) was prepared from the crude mitochondrial fraction, using a 30% Percoll density gradient centrifugation at 95,000× *g* for 30 min at 4 °C. The middle layer that was obtained was re-centrifuged at 9000× *g* for 30 min at 4 °C. The subsequent precipitate was collected as the Mit. The protein concentrations in the S9 and Mit were measured using the BCA kit.

### 4.6. Estimation of Protein Levels of Slc25a39/40 by LC-MS/MS-Based Targeted Proteomics

The protein levels of *Slc25a39/40* in the kidneys of the BDL mice were determined using LC-MS/MS-based targeted proteomics, as described previously [38,39]. Briefly, the proteins of Mit from the kidneys were extracted using the phase-transfer surfactant method. Trypsin was added after the reduction and alkylation of 100 μg of proteins. The surrogate peptides for the target proteins, as shown in Slc25a39: LQSQRPSATSELTTPSR, Slc25a40: FPAPLDMSTWTIMK, and CPS1: IEFEGQSVDFVDPNK, were measured using a liquid chromatographic–tandem mass spectrometry (LC-MS/MS) system (UltiMate 3000 series, Thermo Fisher Scientific, Inc.) and a TSQ Endura Triple Quadrupole Mass Spectrometer with electrospray ionization (Thermo Fisher Scientific, Inc.).

### 4.7. GSH Levels

Measurement of GSH levels in the S9 and Mit was carried out as described in a previous report [40], with minor modifications. The S9 or Mit (100 μg) was filtered using syringe filter units (0.22 μm) and diluted 1:1 with 0.1% formic acid containing 100 ng dopamine as an internal standard. GSH levels in the S9 or Mit were measured using an LC-MS/MS system (UltiMate 3000 series and TSQ Endura Triple Quadrupole Mass Spectrometer). Analysis was performed using a reversed-phase column (COSMOSIL 3PBr [2.0 × 50 mm, 3 μm], Nacalai Tesque, Inc.) with a guard column (AdvanceBio Desalting-RP [2.1 × 12.5 mm], Agilent Technologies, Inc., Santa Clara, CA, USA). The column temperature was set at 35 °C and the autosampler was maintained at 10 °C. The mobile phases were (A) 0.75 mM ammonium formate (pH 3.5) and (B) methanol. Separation was performed under isocratic conditions, with 97% mobile phase A pumped at a flow rate of 0.2 mL/min. The MS scan was operated in the positive ionization mode. The conditions for electrospray ionization were set at 2500 V spray voltage, 280 °C capillary temperature, and 280 °C vaporizer temperature, with 30 arbitrary units of nitrogen sheath gas and 5 arbitrary units of auxiliary gas. The selected reaction monitoring (SRM) mode was used, with argon as the collision gas, at 1.5 mTorr. The mass resolutions were set at 0.7 full width at half height (unit resolution). The SRM transition (precursor and product ions) were the mass-to-charge ratio (m/z) of 308.1 > 76.2, 84.2, 161.9 for GSH, and 154.1 > 137.1 for dopamine. The collision energy was 15 V.

### 4.8. Cell Culture and Treatments

Human clear cell renal carcinoma (KMRC-1) cells were obtained from JCRB Cell Bank (Osaka, Japan). The KMRC-1 cells were maintained in DMEM supplemented with 10% fetal bovine serum, 100 U/mL penicillin, and 100 μg/mL streptomycin. Cells were grown at 37 °C in a humidified incubator equilibrated with 5% CO_2_. The KMRC-1 cells were sub-cultured every 3–4 days using 0.25% trypsin and 1 mM EDTA.

The KMRC-1 cells were seeded at 5 × 10^4^ cells/cm^2^ in 24-well plates (Sumitomo Bakelite Co. Ltd., Tokyo, Japan) 1 day prior to the treatment. The KMRC-1 cells were treated with LPS alone (1 μg/mL) or a combination of LPS-RS (1 μg/mL) as a TLR4 antagonist, FPS-ZM1 (1 μg/mL) as a RAGE antagonist, and LPS (1 μg/mL). The cells were collected using a cell scraper 24 h after the treatment.

### 4.9. Statistical Analysis

Differences in ‘between-group’ means were analyzed using the Bonferroni test after analysis of variance (Figure 7), or unpaired Student’s *t*-test (Figure 1, Figure 4, Figure 5 and Figure 6). GraphPad Prism 5 (GraphPad Software, Inc., La Jolla, CA, USA) was used for all statistical analyses. Statistical significance was set at *p* < 0.05.

## 5. Conclusions

In conclusion, mice with BDL exhibited a decrease in *Slc25a39/40* expression in the kidneys, associated with a reduction in mGSH levels. Mice with LPS-induced inflammation exhibited an increase in Slc25a40 expression in the kidneys, possibly due to the activation of TLR4 and RAGE. To the best of our knowledge, this is the first study that demonstrates the changes in *Slc25a39/40* expression in mice with cholestasis-associated renal injury and LPS-induced inflammation.

## Data Availability

The datasets used and/or analyzed during the current study are available from the corresponding author on reasonable request.

## Supplementary Materials

The information can be downloaded at: https://www.mdpi.com/article/10.3390/ijms23158573/s1.

## Abbreviations

ALT	Alanine aminotransferase;
AST	Aspartate aminotransferase;
BA	Bile acid;
BDL	Bile duct ligation;
DMEM	Dulbecco’s modified Eagle’s medium;
EDTA	Ethylenediaminetetraacetic acid;
GSSG	Glutathione disulfide;
LC-MS/MS	Liquid chromatographic–tandem mass spectrometry;
LPS	Lipopolysaccharide;
mGSH	Mitochondrial GSH;
Mit	Mitochondrial fraction;
m/z	Mass-to-charge ratio;
RAGE	Receptor for advanced glycation end products;
T-BIL	Total bilirubin;
TLR	Toll-like receptor.
