# Supplementary material for: Slc25a39 and Slc25a40 Expression in Mice with Bile Duct Ligation or Lipopolysaccharide Treatment
Source: Int J Mol Sci. 2022 Aug 2;23(15):8573. doi: 10.3390/ijms23158573 (PMC9369313; doi:10.3390/ijms23158573)
Supplement: Supplementary file 1 [file ijms-23-08573-s001.zip › ijms-1809318-supplementary.pdf]

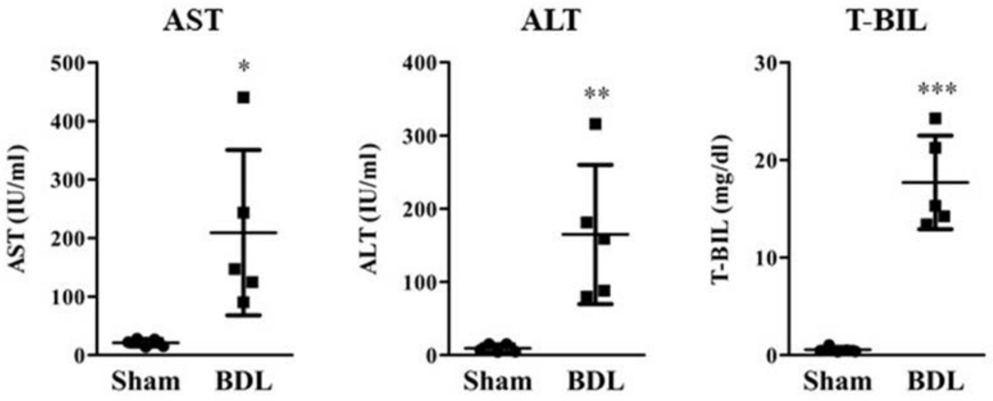

Figure S1. Plasma levels of AST, ALT, and T-BIL in sham and BDL mice. The results are expressed as the mean ± S.D. of each group (n = 5). Significant differences (* $p < 0.05$, ** $p < 0.01$, and *** $p < 0.001$) were observed.
